# A mediation analysis to explain socio‐economic differences in bladder cancer survival

**DOI:** 10.1002/cam4.3418

**Published:** 2020-08-26

**Authors:** Beth Russell, Mieke V. Hemelrijck, Truls Gårdmark, Lars Holmberg, Pardeep Kumar, Andrea Bellavia, Christel Häggström

**Affiliations:** ^1^ Department of Translational Oncology and Urology Research School of Cancer and Pharmaceutical Sciences King's College London London UK; ^2^ Department of Clinical Sciences Danderyd Hospital Karolinska Institute Sweden; ^3^ Department of Surgical Sciences Uppsala University Uppsala Sweden; ^4^ The Royal Marsden NHS Foundation Trust London UK; ^5^ Department of Environmental Health Harvard T.H. Chan School of Public Health; ^6^ Department of Biobank Research Umeå University Umeå Sweden

**Keywords:** bladder cancer, education level, socioeconomic status, survival

## Abstract

**Introduction:**

This study aims to disentangle heterogeneity in the survival of bladder cancer (BC) patients of different socioeconomic status (SES) by identifying potential mediators of the relationship.

**Methods:**

The Bladder Cancer Database Sweden (BladderBaSe) was used to select patients diagnosed between 1997 and 2014 with Tis/Ta‐T4 disease. The education level was used as a proxy for SES. Accelerated failure time models were used to investigate the association between SES and survival. Mediation analysis was used to investigate potential mediators of the association also accounting for interaction.

**Results:**

The study included 37 755 patients from the BladderBaSe. Patients diagnosed with both non‐muscle invasive bladder cancer (NMIBC) and muscle‐invasive bladder cancer (MIBC) who had high SES were found to have increased overall and BC‐specific survival, when compared to those with low SES. In the NMIBC patients, Charlson Comorbidity Index was found to mediate this relationship by 10% (percentage of the total effect explained by the mediator) and hospital type by 4%. The time from referral to TURBT was a considerable mediator (14%) in the MIBC patients only.

**Conclusions:**

Mediation analysis suggests that the association between SES and BC survival can be explained by several factors. The mediators identified were not, however, able to fully explain the theoretical causal pathway between SES and survival, therefore, future studies should also include the investigation of other possible mediators to help explain this relationship further. These results highlight the importance of standardization of clinical care across SES groups.

## Graphical abstract

This is the first time a study has aimed to disentangle the heterogeneity in survival outcomes in bladder cancer by identifying any potential mediators of the relationship between SES and survival. These results highlight the importance of standardization of clinical care across SES groups.

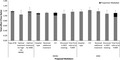

## 1 INTRODUCTION

Bladder cancer (BC) is the 9th most common cancer worldwide with around 550,000 new cases diagnosed in 2018.^1^ There is heterogeneity in the survival of BC patients for many factors such as gender, region, clinical variables, access to care, comorbidity, and risk factors such as smoking and occupational exposure.^2^ The mix of factors associated with survival is complex, and many of these individual factors are associated with socioeconomic status (SES).

Disparities in cancer incidence and mortality have been frequently observed among different socioeconomic groups for several types of cancer including stomach, liver, lips–mouth–pharynx, and lung.^3^, ^4^ A literature review identified that social inequalities in cancer survival are most likely partly attributed to a different stage of disease at diagnosis and access to optimal treatment regimens.^5^ In BC, the link between SES and survival has been extensively studied with disparities in 5‐year survival,^5^ relative risk of death,^6^ and overall survival ^7^ being reported.

Despite this knowledge, there remains a paucity in detailed studies and comprehensive clinical investigations to elucidate the underlying mechanisms behind this association—especially for BC. Mediation analysis can be used to identify factors that are on the causal pathway from the exposure to the outcome, and can partially explain the association.^8^ Therefore, this study aims to ^1^ ascertain a relationship between SES and both overall and BC‐specific survival, and^2^ disentangle the heterogeneity in these survival outcomes by identifying any potential mediators of the relationship.

## 2 METHODS

### 2.1 Data source

2.1

The Bladder Cancer Data Base Sweden (BladderBaSe) was created in 2015. It links information from the Swedish National Register of Urinary Bladder Cancer (SNRUBC) from 1997 to 2014, with a number of national health care and demographic registers through the personal identification numbers.^9^, ^10^

Data from the National Patient Register on discharge diagnoses from hospital admissions up to 10 years prior to the date of BC diagnosis were used to calculate the Charlson Comorbidity Index (CCI) at time of diagnosis, which was categorized into four groups: no comorbidity, 1, 2, and ≥3 comorbidities.^11^, ^12^

### 2.2 Study population and variables

All patients who had been newly diagnosed with BC (Tis, Ta‐T4, any N, any M) between January 1, 1997, and December 31, 2014, were included in the study. Covariates in the study included social and clinical characteristics of the patients such as age, sex, marital status (unmarried, married, divorced, widowed), education level (see below), CCI (0, 1, 2, 3+), diagnosing hospital size (regional, county, other), healthcare region (Stockholm‐Gotland, South, Southeast, Uppsala Örebro, West, North), diagnosis year (1997‐2014), clinical N stage (N0, N+, NX), clinical M stage (M0, M+, MX), WHO grade (G1, G2, G3, GX), date of death and cause of death. Death from BC was defined by the International Classification of Diseases, version 10, code C67.^13^

Education level was used as a proxy measure for SES.^14^, ^15^ Data on the educational level were retrieved from the Longitudinal Integration Database for Health Insurance and Labour Market Studies at Statistics Sweden and categorized into three groups: low (≤9 years of school), medium (10‐12 years), and high (≥13 years), corresponding to mandatory school, high school, and college or university.^16^

### 2.3 Statistical analysis

#### 2.3.1 Survival analysis

Kaplan‐Meier curves, stratified by SES level for both overall and cancer‐specific survival, were first produced to assess the association between SES and overall and BC‐specific survival. Accelerated failure time (AFT) analyses with a Weibull distribution were carried out to produce time ratios (TRs) as a measure of the association between SES and overall and BC‐specific survival. The Weibull distribution was found to be the best fit for the data according to the Akaike's Information Criterion (AIC). The start date of the study was the date of diagnosis and the last date of follow‐up was the date of death, emigration, or December 31, 2014, whichever occurred first. Time in years from diagnosis was used as the timescale. All models were adjusted for age (continuous), sex, CCI, marital status, healthcare region, hospital type, diagnosis year, clinical N stage, clinical M stage, and WHO grade.

#### 2.3.2 Mediation analysis

**Figure 1:**
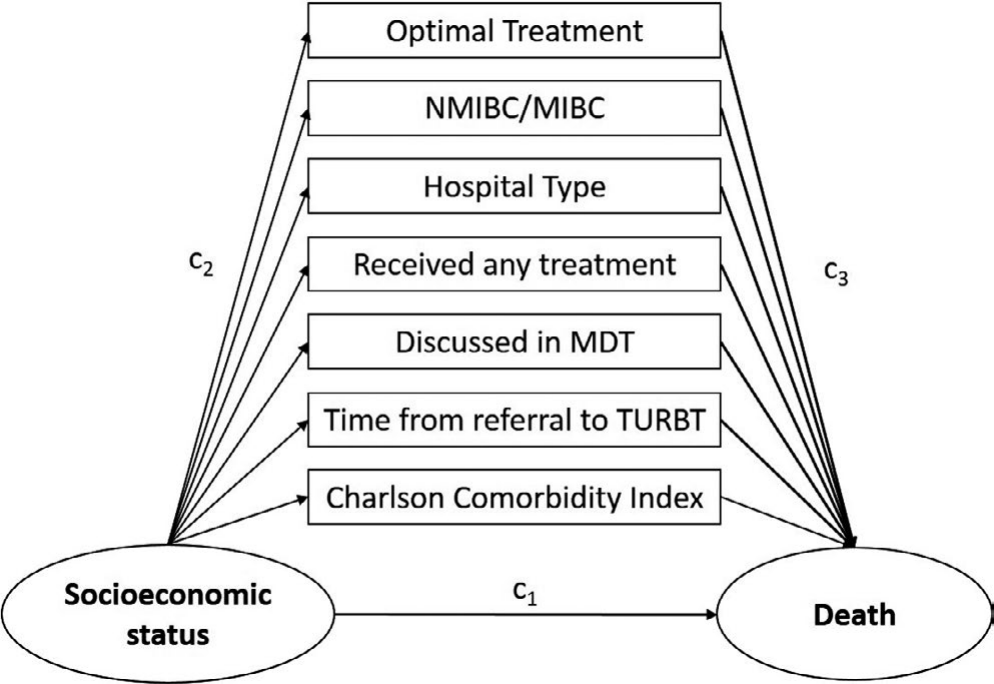
Directed Acyclic Graph for Mediation Analysis Model. c1 – confounders of the exposure‐outcome relationship; c2 – confounders of the exposure‐mediator relationship; c3 ‐ confounders of the mediator‐outcome relationship. NMIBC ‐ Non‐muscle invasive bladder cancer; MIBC ‐ Muscle‐invasive bladder cancer; MDT ‐ Multi‐disciplinary team; TURBT ‐ Transurethral resection of the bladder tumor

We hypothesized mediators for the causal pathway between SES and survival as depicted in Figure 1.

The mediators initially investigated were: type of bladder cancer (NMIBC/MIBC) and optimal treatment for high‐risk NMIBC/MIBC (yes/no). We looked at these mediators first to examine whether to stratify by NMIBC/MIBC in the next set of analyses. The mediators subsequently investigated in subgroup analyses for NMIBC (Tis, Ta‐T1) and MIBC (T2‐T4) were: hospital type (county, other/regional), received additional primary treatment (yes/no), discussed in multidisciplinary team (MDT) meeting (yes/no), time from referral to transurethral resection of the bladder tumor (TURBT) (≤12 days/>12 days) and CCI (0/1+).

Optimal treatment was defined as: intravesical treatment with BCG or radical cystectomy for those with high‐risk NMIBC, and as radical cystectomy (if diagnosed before 2008) or neoadjuvant chemotherapy plus radical cystectomy (if diagnosed after 2008) or radical radiotherapy for those with MIBC. Those with a missing cystectomy date (n = 179) were given an estimated date by calculating their diagnosis date plus 102 days, which is the median number of days between diagnosis and cystectomy in the BladderBaSe in 2014 (Q1 to Q3: 75 to 144 days).

Received additional primary treatment was defined as instillation of Bacillus Calmette‐Guérin (BCG), Mitomycin‐C (not the immediately post‐operative dose), external radiation therapy, chemotherapy or radical cystectomy.

For the mediation analysis, several different sub‐populations were selected depending on the mediator being investigated. These were:

- All patients diagnosed between 1997 and 2014 and…:
  - Tis, Ta‐T1 (NMIBC)
  - T2‐T4 (MIBC)
  - TaG3‐T1G2G3/Cis, N0, M0 (High‐risk NMIBC)
  - T2‐T4, N0, M0 (MIBC with no metastasis)
- All patients diagnosed 2008 onwards (as this is when the referral date and MDT information was available from) with any M or N stage and…:
  - Tis, Ta‐T1 (NMIBC)
  - T2‐T4 (MIBC)
  - T1 only (NMIBC) (only T1 patients are discussed in MDT meeting)

Mediation analysis was performed using the “med4way” command in STATA.^17^, ^18^ This method decomposes the total effect (TE, the effect of SES on overall survival) into four components: controlled direct effect (CDE, the effect neither due to the mediator nor to exposure‐mediator interaction), reference interaction (INTref, the effect only due to interaction), mediated interaction (INTmed, the effect due to interaction only active when mediation is present) and the pure indirect effect (PIE, the effect due to mediation alone). The decomposition can be explained by the following equation:^18^

TE=CDE+INTref+INTmed+PIE

The proportion mediated by each mediator was also calculated. We fitted the outcome (overall survival) with an AFT regression assuming a Weibull distribution, and fitted a logistic regression model for the mediator. For the mediation analysis, SES was categorized into two groups: low = low and high = medium/high. The same variables were adjusted for in the mediation models as above in the AFT models. However, when optimal treatment was analyzed as a mediator, the models were not adjusted for N or M stage as these were already limited. These covariates were selected when considering all possible confounders (c_1_, c_2_, and c_3_) of the mediated relationship as depicted in Figure 1.

### 2.4 Sensitivity analyses

Sensitivity analyses for unmeasured confounding from smoking status were subsequently carried out by producing a bias factor using the following equation (8):

$$Biasfactor=\frac{1+\left(\gamma ‐1\right)P\left(U=1|a_{1},c\right)}{1+\left(\gamma ‐1\right)P\left(U=1|a_{0},c\right)}$$

where U = smoking status; γ = the effect of smoking on the outcome; P (U = 1|a1, c) = prevalence of smoking in the exposed; P (U = 1|a0, c) = prevalence of smoking in the unexposed.

Further sensitivity analyses were carried out on the mediation models whereby the level at which the mediator was set in the model was changed.

All data management and statistical analyses were performed with STATA MP/2 version 14 (StataCorp LP, College Station, Texas).

## 3 RESULTS

### 3.1 Demographics

About 37,755 patients were identified as having Tis, Ta‐T4 disease (74% NMIBC and 26% MIBC) (Table 1). About 49% of patients had a low level of SES, 35% had a medium level of SES whilst 16% had a high level of SES. The median survival time for all patients was 3.44 years (IQR: 1.14‐7.59), 4.60 years for NMIBC patients (IQR:1.92‐8.63), and 1.05 years for MIBC patients (IQR:0.42‐3.01).

**Table 1:** Cohort Characteristics, Overall, and when Stratified by SES

Variable	Overall	SES		
		Low	Medium	High
	N (%)	N (%)	N (%)	N (%)
SES	37,755 (100.00)	18,473 (100.00)	13,110 (100.00)	6172 (100.00)
Age at diagnosis				
<50	1202 (3.18)	269 (1.46)	600 (4.59)	333 (5.40)
50‐59	3679 (9.74)	1094 (5.92)	1658 (12.65)	927 (15.02)
60‐69	9382 (24.85)	3769 (20.40)	3742 (28.54)	1871 (30.31)
70‐79	13,163 (34.86)	6864 (37.16)	4347 (33.16)	1952 (31.63)
80‐89	9165 (24.27)	5634 (30.50)	2540 (19.37)	991 (16.06)
≥90	1164 (3.08)	843 (4.56)	223 (1.70)	98 (1.59)
Sex				
Male	28,159 (74.58)	13,525 (73.21)	9901 (75.52)	4733 (76.69)
Female	9596 (25.42)	4948 (26.79)	3209 (24.48)	1439 (23.31)
CCI				
0	22,265 (58.97)	10,080 (54.57)	8077 (61.61)	4108 (66.56)
1	6416 (16.99)	3483 (18.85)	2095 (15.98)	838 (13.58)
2	5070 (13.43)	2667 (14.44)	1636 (12.48)	767 (12.43)
3+	4004 (10.61)	2243 (12.14)	1302 (9.93)	459 (7.44)
Marital Status				
Unmarried	3667 (9.71)	1790 (9.69)	1324 (10.10)	553 (8.96)
Married	21,550 (57.08)	9868 (53.42)	7728 (58.95)	3954 (64.06)
Divorced	5439 (14.41)	2374 (12.85)	2136 (16.29)	929 (15.05)
Widowed	6723 (17.81)	4216 (22.82)	1850 (14.11)	657 (10.64)
Missing	376 (1.00)	225 (1.22)	72 (0.55)	79 (1.28)
Clinical T stage				
Tis	1035 (2.74)	451 (2.44)	385 (2.94)	199 (3.22)
Ta	18,354 (48.61)	8398 (45.46)	6629 (50.56)	3327 (53.90)
T1	8557 (22.66)	4192 (22.69)	3005 (22.92)	1360 (22.03)
T2	6545 (17.34)	3462 (18.74)	2165 (16.51)	918 (14.87)
T3	1989 (5.27)	1220 (6.60)	543 (4.14)	226 (3.66)
T4	1275 (3.38)	750 (4.06)	383 (2.92)	142 (2.30)
N stage				
N0	10,668 (28.26)	4748 (25.70)	3972 (30.30)	1948 (31.56)
N+	1315 (3.48)	690 (3.74)	461 (3.52)	164 (2.66)
NX	25,583 (67.76)	12,941 (70.05)	8617 (65.73)	4025 (65.21)
Missing	189 (0.50)	94 (0.51)	60 (0.46)	35 (0.57)
M stage				
M0	10,647 (28.20)	5121 (27.72)	3785 (28.87)	1741 (28.21)
M+	1179 (3.12)	668 (3.62)	376 (2.87)	135 (2.19)
MX	25,590 (67.78)	12,526 (67.81)	8832 (67.37)	4232 (68.57)
Missing	339 (0.90)	158 (0.86)	117 (0.89)	64 (1.04)
WHO grade				
G1	9806 (25.97)	4358 (23.59)	3614 (27.57)	1834 (29.71)
G2	12,050 (31.92)	5961 (32.27	4153 (31.68)	1936 (31.37)
G3	14,794 (39.18)	7585 (41.06)	4983 (38.01)	2226 (36.07)
GX	444 (1.18)	244 (1.32)	137 (1.05)	63 (1.02)
Missing	661 (1.75)	325 (1.76)	223 (1.70)	113 (1.83)
Hospital type				
Regional	11,686 (30.95)	5085 (27.53)	4299 (32.79)	2302 (37.30)
County	17,266 (45.73)	8631 (46.72)	5970 (45.54)	2665 (43.18)
Other	8803 (23.32)	4757 (25.75)	2841 (21.67)	1205 (19.52)
Healthcare Region				
Stockholm‐Gotland	6726 (17.81)	2590 (14.02)	2612 (19.92)	1524 (24.69)
South	8096 (21.44)	3987 (21.58)	2755 (21.01)	1354 (21.94)
Southeast	4420 (11.71)	2391 (12.94)	1449 (11.05)	580 (9.40)
Uppsala Örebro	8007 (21.21)	4214 (22.81)	2649 (20.21)	1144 (18.54)
West	6984 (18.50)	3533 (19.13)	2346 (17.89)	1105 (17.90)
North	3518 (9.32)	1757 (9.51)	1296 (9.89)	465 (7.53)
Missing	4 (0.01)	1 (0.01)	3 (0.02)	0 (0.00)
Year of diagnosis				
1997‐2007	21,307 (56.43)	11,376 (61.58)	6904 (52.66)	3027 (49.04)
2008‐2014	16,448 (43.57)	7097 (38.42)	6206 (47.34)	3145 (50.96)

Note

Those with a missing value for education were entered into the “low” category (N = 1292). SES – socioeconomic status; CCI – Charlson comorbidity index; WHO: world health organization

### 3.2 Overall and bladder cancer‐specific survival

**Figure 2:**
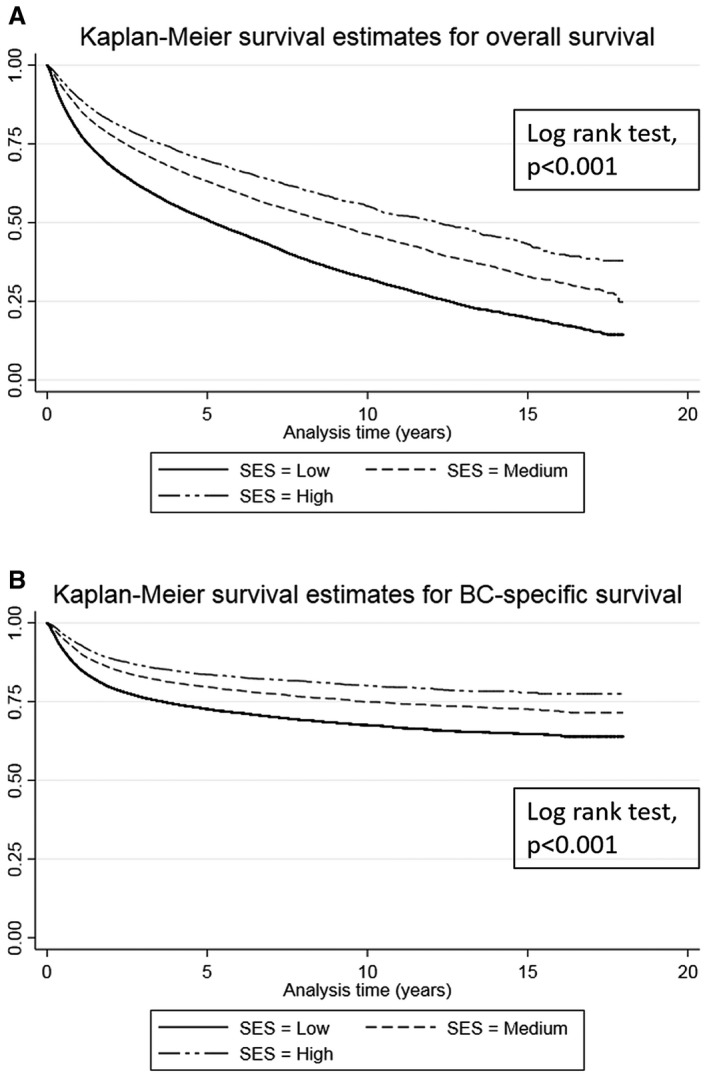
Kaplan Meier Curves for Overall and Bladder Cancer‐Specific Survival when Stratified by SES A) Overall survival, B) bladder cancer‐specific survival. SES – socioeconomic status; BC – bladder cancer

Figure 2 shows Kaplan‐Meier survival estimates of these relationships. Patients diagnosed with NMIBC and MIBC who had a medium or high SES were found to be associated with longer survival from both any cause and BC‐specific death (Table 2) when compared to those with a low SES. Overall survival: NMIBC, medium and high SES, TR = 1.09, 95% CI: 1.06, 1.13 and TR = 1.22, 95% CI: 1.16, 1.29, respectively. MIBC, medium and high SES, TR = 1.16, 95% CI: 1.08, 1.24 and TR = 1.37, 95% CI: 1.24, 1.51, respectively. Table 2 shows that the results for BC‐specific death show a similar pattern.

**Table 2:** Accelerated Failure Time Models Looking at the Association Between SES and Overall and Bladder Cancer‐Specific Survival

	N/total	Overall Survival				Bladder Cancer‐Specific Survival			
		TR^a^	95% CI	TR^b^	95% CI	TR^a^	95% CI	TR^b^	95% CI
NMIBC									
SES									
Low	13,041/27,946	1.00	Ref.	1.00	Ref.	1.00	Ref.	1.00	Ref.
Medium	10,019/27,946	1.49	1.43, 1.55	1.09	1.06, 1.13	1.52	1.37, 1.69	1.11	1.01, 1.22
High	4886/27,946	1.93	1.82, 2.05	1.22	1.16, 1.29	2.11	1.81, 2.46	1.34	1.17, 1.53
MIBC									
SES									
Low	5432/9809	1.00	Ref.	1.00	Ref.	1.00	Ref.	1.00	Ref.
Medium	3091/9809	1.56	1.45, 1.68	1.16	1.08, 1.24	1.49	1.36, 1.64	1.15	1.06, 1.26
High	1286/9809	2.14	1.92, 2.39	1.37	1.24, 1.51	2.10	1.83, 2.42	1.40	1.24, 1.59

Note

TR^a^ – unadjusted model, TR^b^ ‐ adjusted for CCI, age, marital status, sex, healthcare region, hospital type, diagnosis year, M stage, N stage, and grade. NMIBC – non‐muscle‐invasive bladder cancer; MIBC‐ muscle‐invasive bladder cancer; SES – socioeconomic status

### 3.3 Mediation analysis

#### 3.3.1 Type of bladder cancer

**Figure 3:**
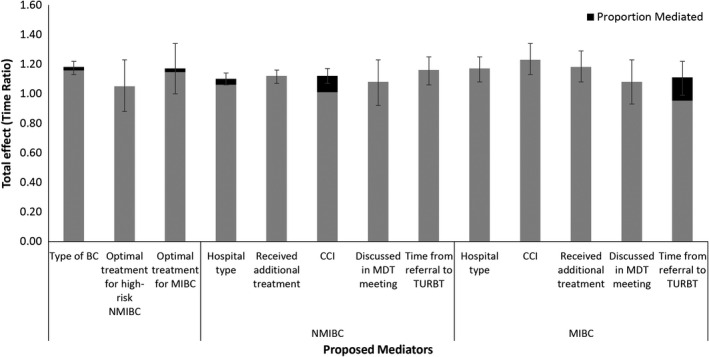
Total Effect (with 95% CIs) and Proportion of Each Total Effect Mediated by Each Proposed Mediator. BC: bladder cancer; NMIBC: non‐muscle invasive bladder cancer; MIBC: muscle‐invasive bladder cancer; CCI: Charlson Comorbidity Index; MDT: multi‐disciplinary team; TURBT: transurethral resection of the bladder tumor

When investigating the type of bladder cancer (NMIBC vs MIBC) as a possible mediator (Table 3), patients who had a higher SES were more likely to be diagnosed with NMIBC compared to patients with a lower SES (OR = 0.82, 95% CI: 0.78, 0.86). The four‐way decomposition revealed a total effect of 18% longer survival (TR = 1.18; 95% CI: 1.13, 1.22) and the proportion of this association mediated by bladder cancer type was 2% (Figure 3).

**Table 3:** Mediation Analysis and Four‐way Decomposition

Cohort	Mediator		N/total	Model for outcome (TR)	95% CI	Model for mediator (OR)	95% CI	4 way decomposition					Proportion mediated (%)
								Total effect (TR)	95% CI	Proportion due to neither mediation nor interaction (CDE) (%)	Proportion due to interaction (%)	Proportion due to indirect effect (IE) (%)	
Tis, Ta‐T4, any M, any N stage	Type of bladder cancer	NMIBC	27,946/37,755	1.17	1.13, 1.22	0.82	0.78, 0.86	1.18	1.13, 1.22	17.52	80.92	1.56	1.87
		MIBC*	9809/37,755										
Tis, Ta‐T1, M0, N0	Optimal treatment for high‐risk NMIBC	Yes*	991/2049	1.05	0.90, 1.25	1.26	1.03, 1.54	1.05	0.88, 1.23	614.27	‐514.27	0.00	0.00
		No	1058/2049										
T2‐T4, M0, N0	Optimal treatment for MIBC	Yes*	1475/3027	0.97	0.84, 1.13	1.11	0.93, 1.31	1.17	1.00, 1.34	106.53	‐7.79	1.27	2.02
		No	1552/3027										

Note

* Level at which the mediator was set for the mediation analysis; CDE, controlled direct effect; IE, indirect effect; Int Med, mediated interaction; Int Ref, reference interaction; MIBC, muscle‐invasive bladder cancer; NMIBC, non‐muscle‐invasive bladder cancer; OR, odds ratio; TR, time ratio. All analyses are results for those with medium/high education compared to a low education (reference).

#### 3.3.2 Optimal treatment

2049 high‐risk NMIBC (TaG3‐T1G2G3/Cis, N0, M0) and 3027 non‐metastatic MIBC patients (T2‐T4, N0, M0) were identified (Table 3). Patients with high‐risk NMIBC and a high SES were more likely to receive optimal treatment compared to those with a low SES (OR:1.26, 95% CI: 1.03, 1.54). The proportion mediated by receiving optimal treatment was calculated to be 0% (Figure 3).

#### 3.3.3 Hospital type

A higher SES was found to be negatively associated with patients being treated at a regional hospital in both NMIBC and MIBC patients (OR: 0.79, 95% CI: 0.74, 0.84, and OR: 0.77, 95% CI: 0.70, 0.85, respectively) (Table 4). The association between SES and overall survival was found to be 4% mediated by hospital type in NMIBC and 0% in MIBC patients (Figure 3).

**Table 4:** Mediation Analysis when Cohort is Stratified by NMIBC and MIBC

Cohort	Mediator		N/total	Model for outcome (TR)	95% CI	Model for mediator (OR)	95% CI	4 way decomposition					
								Total effect (TR)	95% CI	Proportion due to neither mediation nor interaction (CDE) (%)	Proportion due to interaction (%)	Proportion due to indirect effect (IE) (%)	Proportion mediated (%)
NMIBC													
Tis, Ta‐T1, any M, any N stage	Hospital type	Regional*	8388/27,946	1.11	1.04, 1.17	0.79	0.74, 0.84	1.10	1.06, 1.14	106.11	‐8.66	2.56	3.55
		County/ Other	19,558/27,946										
Tis, Ta‐T1, any M, any N stage	Received additional treatment	Yes*	6926/27,946	1.12	1.08, 1.16	1.08	1.01, 1.15	1.12	1.07, 1.16	17.43	82.47	0.10	‐0.18
		No	20,494/27,946										
Tis, Ta‐T1, any M, any N stage	Charlson Comorbidity Index	0*	16,724/27,946	1.11	1.06, 1.17	0.82	0.78, 0.87	1.12	1.07, 1.17	98.79	‐7.31	8.52	9.83
		≥1	11,222/27,946										
T1, any M, any N stage and diagnosed after 2008	Discussed in MDT meeting	Yes*	1498/4258	1.23	1.00, 1.51	0.89	0.76, 1.04	1.08	0.92, 1.23	99.33	1.04	‐0.37	‐0.26
		No	2703/4258										
Tis, Ta‐T1, any M, any N stage and diagnosed after 2008	Time from referral to TURBT	≤12 days*	1061/12,531	1.08	0.90, 1.31	1.06	0.92, 1.21	1.16	1.06, 1.25	29.97	70.03	0.00	0.00
		>12 days	10,845/12,531										
MIBC													
T2‐T4, any M, any N stage	Hospital type	County/Other	3298/9809	1.20	1.07, 1.32	0.77	0.70, 0.85	1.17	1.08, 1.25	123.09	‐23.09	0.00	0.00
		Regional*	6511/9809										
T2‐T4, any M, any N stage	Received additional treatment	Yes*	5266/9809	1.13	1.03, 1.22	1.17	1.05, 1.31	1.18	1.08, 1.29	100.00	0.00	0.00	0.00
		No	4387/9809										
Tis, Ta‐T1, any M, any N stage	Charlson Comorbidity Index	0*	5541/9809	1.24	1.14, 1.34	0.89	0.81, 0.97	1.23	1.13, 1.34	100.00	0.00	0.00	0.00
		≥1	4268/9809										
T2‐T4, any M, any N stage and diagnosed after 2008	Discussed in MDT meeting	Yes*	1681/3917	1.07	0.48, 1.22	0.94	0.80, 1.09	1.08	0.93, 1.23	100.00	0.00	0.00	0.00
		No	2191/3917										
T2‐T4, any M, any N stage and diagnosed after 2008	Time from referral to TURBT	≤12 days*	704/3917	0.96	0.78, 1.17	1.23	1.03, 1.48	1.11	0.99, 1.22	‐25.81	115.39	10.42	14.18
		>12 days	3021/3917										

Note

*Level at which the mediator was set for the mediation analysis; All analyses are results for those with medium/high education compared to a low education (reference); CDE, controlled direct effect; IE, indirect effect; Int Med, mediated interaction; Int Ref, reference interaction; MDT, multidisciplinary team; MIBC, muscle‐invasive bladder cancer; NMIBC, non‐muscle‐invasive bladder cancer; OR, odds ratio; TR, time ratio; TURBT, trans‐urethral resection of the bladder tumor.

#### 3.3.4 Received additional treatment

Patients with a higher SES were positively associated with having received additional treatment for their bladder cancer compared to patients with a lower SES for both NMIBC and MIBC patients (OR: 1.08, 95% CI: 1.01, 1.15 and OR: 1.17, 95% CI: 1.05, 1.31, respectively) (Table 4). However, receiving additional treatment was not found to be a mediator to the association between SES and survival (Figure 3).

#### 3.3.5 Time from referral to TURBT

A higher SES was positively associated with having a longer time (>12 days) between referral to TURBT in MIBC patients (Table 4). This relationship was 14% mediated by the time between referral and TURBT in MIBC patients and 0% in NMIBC patients (Figure 3).

#### 3.3.6 Charlson comorbidity index

A higher SES was associated with lower odds of having a CCI score of at least 1 in both NMIBC and MIBC (Table 4). The TE for NMIBC was TR = 1.12 (95% CI: 1.07, 1.17) and this was found to be 10% mediated by CCI (Figure 3). Conversely, CCI was not found to be a mediator between SES and overall survival in MIBC patients.

### 3.4 Sensitivity analyses

Data from the Swedish Public Health Authority were used to estimate the prevalence of smoking as 19.5% and 12% in the low and high education groups, respectively.^19^ The effect of smoking on the outcome was estimated to be 1.20 and 1.09 for overall and cancer‐specific mortality, respectively.^20^ Subsequently,

the bias factor was calculated as 0.99 for both overall and cancer‐specific mortality. Therefore, the survival analysis, prior to mediation analysis was not changed significantly by additionally controlling for unmeasured confounding from smoking status.

The sensitivity analyses in Tables S1 and S2 show the results when changing the level at which the mediator was set. Differences in the proportions contributed to by the CDE and reference interaction were found, but did not show any differences in the TE or the proportion mediated by any of the hypothesized mediators.

## 4 DISCUSSION

This large observational study using mediation analysis revealed that the relationship between SES and survival is explained by a variety of factors: for example, hospital type and CCI in NMIBC and a delay in time between referral and TURBT in MIBC patients. Furthermore, a higher SES was associated with several factors including being treated at a county/other hospitals (NMIBC and MIBC); receiving treatment for their NMIBC or MIBC; and having a delay of less than 12 days from referral to TURBT in MIBC.

Patients with high‐risk NMIBC and higher SES were found to be more likely to receive optimal treatment. Previous studies have suggested that the lower SES groups may have a different understandings or beliefs about treatment.^21^ Furthermore, racial disparities in treatments have been seen in MIBC before.^22^ Receiving optimal treatment was only found to be a mediator on the relationship between SES and survival in the MIBC patients, though the proportion mediated was minor (2%). This point is important for clinicians as it suggests that patients across different SES groups are not always being offered a consistent level of care and this inconsistency may be impacting the survival of MIBC patients.

A study by Begum et al did not see any differences in treatments or delays between the different socioeconomic groups.^7^ In the current study, however, we found that patients with a higher SES had higher odds of receiving additional treatment for their malignancy in both NMIBC and MIBC. This result is similar to the study by Klapheke and colleagues whereby they found patients with a lower SES were significantly less likely to accept chemotherapy for their metastatic BC.^23^ In the current study, MIBC patients with a higher SES had 23% increased odds of experiencing a delay between referral and TURBT compared to those with a lower SES thus differing from Begum's study. The total effect of SES on survival was subsequently 14% mediated by this delay. This topic is of particular interest as it has recently been reported in the press that BC patients often experience delays to definitive treatment.^24^ It is important that clinicians are aware of the discrepancies in delay time among different SES groups and that this is having a substantial impact on survival.

SES can be quantified in many different ways including education level, home owner status, salary or type of worker (eg blue‐collar, white collar or self‐employed), all of which are correlated to varying degrees.^25^ The existing studies investigating SES and survival for BC have so far investigated discrepancies in survival for patients in relation to annual salaries, those living in deprived countries/postcodes, and marital status.^26^, ^27^, ^28^ However, to the best of our knowledge, this is the first study to ascertain education as a prognostic factor relating to SES in BC patients and our results are in concordance with other SES quantifiers. Whilst all SES indicators have their own advantages, education captures aspects of their social opportunities as well as future employment and income.^25^ Nonetheless, we were unable to consider age and gender as possible mediators since education may be an unreliable proxy for SES for these variables. This is because, in the older generation, women may not be well educated but may have a high SES due to marrying a wealthy spouse.

To the best of our knowledge, this study is the first of its kind to use mediation analysis to explore the relationship between SES and survival in BC patients. The main strength of the study was the use of a large nationwide database with more than 15 years of follow‐up. The real‐world nature of the data means it exhibits high external validity.^29^ However, its main strength also comes limitations inherent to the use of observational data such as the paucity of information on certain variables. In the current study, the lack of information and thus adjustment for smoking status was a disadvantage. Despite this, sensitivity analyses suggested our survival analyses were not sensitive to unmeasured confounding from smoking. There was also limited detailed information on the surgical and systemic treatment variables (eg surgery type or dose of chemotherapy).

Further disadvantages to this study include the lack of validation of the clinical data from the SNRUBC and the use of one model for each mediator. Future studies could consider integrating all possible mediators into one multiple‐mediators model.

Many survival studies choose to use Cox proportional hazards models. However, as the Cox model assumes a rare outcome when used in mediation analysis, AFT models were more appropriate for this study. When the survival times follow a Weibull distribution, AFT and Cox models can be used interchangeably; it is the interpretation of the results which differ significantly.^30^ HRs, which are the output of a Cox model, estimate the relative risk of death whilst TRs, the output of AFT models, estimate the change in mean survival time.^30^ The main assumption we make in the AFT analyses is that survival times follow a Weibull distribution, which implies that the effect of the exposure on survivorship is roughly consistent throughout the lifespan.^31^

## 5 CONCLUSION

This study is the first of its kind to attempt to delve into the factors behind the association between SES and survival. Mediation analysis suggested that the hypothesized relationship between SES and survival was contributed to by several factors with some being avoidable, for example, CCI and a delay in time between referral and TURBT, whilst others such as hospital type are less manageable. SES was also associated with many clinical factors thereby highlighting the importance of standardization of clinical care across SES groups.

## CONFLICTS OF INTEREST

There are no conflicts of interest to declare.

## AUTHOR CONTRIBUTION

Study design – BR, MVH, TG, LH, PK, AB, and CH. Data analysis – BR and CH. Writing and review of the manuscript ‐ BR, MVH, TG, LH, PK, AB, and CH.

## Supporting information

Table S1‐2

## Data Availability

The BladderBaSe data is held on a secure server and is therefore not publically available. However, applications to access the data can be made by contacting support.rcnorr@vll.se.
