# Supplementary material for: A mediation analysis to explain socio‐economic differences in bladder cancer survival
Source: Cancer Med. 2020 Aug 26;9(20):7477–87. doi: 10.1002/cam4.3418 (PMC7571835; doi:10.1002/cam4.3418)
Supplement: Supplementary file 1 — Table S1‐2 [file CAM4-9-7477-s001.docx]

**Supplementary Tables**

**Supplementary Table 1. Mediation Analysis and Four-way Decomposition (Mediator Reference Level Changed)**

| **Cohort** | **Mediator** | | **N/total** | **Model for outcome (TR)** | **95% CI** | **Model for mediator (OR)** |  | **4 way decomposition** | | | | | |
| --- | --- | --- | --- | --- | --- | --- | --- | --- | --- | --- | --- | --- | --- |
|  |  |  |  |  |  |  | **95% CI** | **Total effect (TR)** | **95% CI** | **Proportion due to neither mediation nor interaction (CDE) (%)** | **Proportion due to interaction (%)** | **Proportion due to indirect effect (IE) (%)** | **Proportion mediated (%)** |
| Tis, Ta-T1, M0, N0 | Optimal treatment for high-risk NMIBC | Yes | 991/2049 | 1.05 | 0.90, 1.25 | 1.26 | 1.03, 1.54 | 1.05 | 0.88, 1.23 | 100.00 | 0.00 | 0.00 | 0.00 |
|  |  | No* | 1058/2049 |  |  |  |  |  |  |  |  |  |  |
| T2-T4, M0, N0 | Optimal treatment for MIBC | Yes | 1475/3027 | 0.97 | 0.84, 1.13 | 1.11 | 0.93, 1.31 | 1.17 | 1.00, 1.34 | -10.37 | 109.10 | 1.27 | 2.02 |
|  |  | No* | 1552/3027 |  |  |  |  |  |  |  |  |  |  |
| Tis, Ta-T4, any M, any N stage | Type of bladder cancer | NMIBC* | 27,946/37,755 | 1.17 | 1.13, 1.22 | 0.82 | 0.78, 0.86 | 1.18 | 1.13, 1.22 | 99.88 | -1.44 | 1.56 | 1.87 |
|  |  | MIBC | 9809/37,755 |  |  |  |  |  |  |  |  |  |  |

*CDE, controlled direct effect; IE, indirect effect; Int Med, mediated interaction; Int Ref, reference interaction; MIBC, muscle invasive bladder cancer; NMIBC, non-muscle invasive bladder cancer; OR=odds ratio; TR=time ratio. All analyses are results for those with medium/high education compared to a low education (reference); * Level at which the mediator was set for the mediation analysis.*

**Supplementary Table 2. Mediation Analysis When Cohort is Stratified by NMIBC and MIBC: Sensitivity Analysis When the Reference for the Mediator is Changed**

| **Cohort** | **Mediator** | | **N/total** | **Model for outcome (TR)** | **95% CI** | **Model for mediator (OR)** | **95% CI** | **4 way decomposition** | | | | | |
| --- | --- | --- | --- | --- | --- | --- | --- | --- | --- | --- | --- | --- | --- |
|  |  |  |  |  |  |  |  | **Total effect (TR)** | **95% CI** | **Proportion due to neither mediation nor interaction (CDE) (%)** | **Proportion due to interaction (%)** | **Proportion due to indirect effect (IE) (%)** | **Proportion mediated (%)** |
| **NMIBC** | | | | | | | | | | | | | |
| Tis, Ta-T1, any M, any N stage | Hospital type | Regional | 8388/27,946 | 1.11 | 1.04, 1.17 | 0.79 | 0.74, 0.84 | 1.10 | 1.06, 1.14 | 89.30 | 8.15 | 2.56 | 3.55 |
|  |  | County/ Other* | 19,558/27,946 |  |  |  |  |  |  |  |  |  |  |
| Tis, Ta-T1, any M, any N stage | Received  additional treatment | Yes | 6926/27,946 | 1.12 | 1.08, 1.16 | 1.08 | 1.01, 1.15 | 1.12 | 1.07, 1.16 | 103.83 | -3.95 | 0.10 | -0.18 |
|  |  | No* | 20,494/27,946 |  |  |  |  |  |  |  |  |  |  |
| Tis, Ta-T1, any M, any N stage | Charlson Comorbidity Index | 0 | 16,724/27,946 | 1.11 | 1.06, 1.17 | 0.82 | 0.78, 0.87 | 1.12 | 1.07, 1.17 | 43.70 | 47.78 | 8.52 | 9.83 |
|  |  | ≥1* | 11,222/27,946 |  |  |  |  |  |  |  |  |  |  |
| Tis, Ta-T1, any M, any N stage and diagnosed after 2008 | Discussed in MDT meeting | Yes | 1498/4258 | 1.20 | 0.98, 1.48 | 0.88 | 0.76, 1.03 | 1.07 | 0.92, 1.22 | 215.97 | -115.81 | -0.40 | -0.28 |
|  |  | No* | 2703/4258 |  |  |  |  |  |  |  |  |  |  |
| Tis, Ta-T1, any M, any N stage and diagnosed after 2008 | Time from referral to TURBT | ≤12 days | 1061/12,531 | 1.08 | 0.90, 1.31 | 1.06 | 0.92, 1.21 | 1.16 | 1.06, 1.25 | 100.00 | 0.00 | 0.00 | 0.00 |
|  |  | >12 days* | 10,845/12,531 |  |  |  |  |  |  |  |  |  |  |
| **MIBC** | | | | | | | | | | | | | |
| T2-T4, any M, any N stage | Hospital type | Regional | 3298/9809 | 1.20 | 1.07, 1.32 | 0.77 | 0.70, 0.85 | 1.17 | 1.08, 1.25 | 100.00 | 0.00 | 0.00 | 0.00 |
|  |  | County/  Other* | 6511/9809 |  |  |  |  |  |  |  |  |  |  |
| T2-T4, any M, any N stage | Received  additional treatment | Yes | 5266/9809 | 1.13 | 1.03, 1.22 | 1.17 | 1.05, 1.31 | 1.18 | 1.08, 1.29 | 32.70 | 67.30 | 0.00 | 0.00 |
|  |  | No* | 4387/9809 |  |  |  |  |  |  |  |  |  |  |
| T2-T4, any M, any N stage | Charlson Comorbidity Index | 0 | 5541/9809 | 1.24 | 1.14, 1.34 | 0.89 | 0.81, 0.97 | 1.23 | 1.13, 1.34 | 27.58 | 72.42 | 0.00 | 0.00 |
|  |  | ≥1* | 4268/9809 |  |  |  |  |  |  |  |  |  |  |
| T2-T4, any M, any N stage and diagnosed after 2008 | Discussed in MDT meeting | Yes | 1681/3917 | 1.27 | 1.08, 1.48 | 0.99 | 0.89, 1.09 | 1.11 | 1.02, 1.21 | 173.27 | 73.27 | 0.00 | 0.00 |
|  |  | No* | 2191/3917 |  |  |  |  |  |  |  |  |  |  |
| T2-T4, any M, any N stage and diagnosed after 2008 | Time from referral to TURBT | ≤12 days | 704/3917 | 0.96 | 0.78, 1.17 | 1.23 | 1.03, 1.48 | 1.11 | 0.99, 1.22 | 109.04 | -22.84 | 10.42 | 14.18 |
|  |  | >12 days* | 3021/3917 |  |  |  |  |  |  |  |  |  |  |

*CDE, controlled direct effect; IE, indirect effect; Int Med, mediated interaction; Int Ref, reference interaction; MDT, multidisciplinary team; MIBC, muscle invasive bladder cancer; NMIBC, non-muscle invasive bladder cancer; OR=odds ratio; TURBT, trans-urethral resection of the bladder tumour; TR=time ratio; *Level at which the mediator was set for the mediation analysis; All analyses are results for those with medium/high education compared to a low education (reference).*
